# Blastocyst genotyping for quality control of mouse mutant archives: an ethical and economical approach

**DOI:** 10.1007/s11248-015-9897-1

**Published:** 2015-07-16

**Authors:** Ferdinando Scavizzi, Edward Ryder, Stuart Newman, Marcello Raspa, Diane Gleeson, Hannah Wardle-Jones, Lluis Montoliu, Almudena Fernandez, Marie-Laure Dessain, Vanessa Larrigaldie, Zuzana Khorshidi, Reetta Vuolteenaho, Raija Soininen, Philippe André, Sylvie Jacquot, Yi Hong, Martin Hrabe de Angelis, Ramiro Ramirez-Solis, Brendan Doe

**Affiliations:** Consiglio Nazionale delle Ricerche (IBCN), CNR-Campus International Development (EMMA-INFRAFRONTIER- IMPC), A. Buzzati-Traverso Campus, Via E. Ramarini 32, 00015 Monterotondo Scalo, Roma Italy; Wellcome Trust Sanger Institute, Hinxton, Cambridgeshire CB10 1SA UK; Department of Molecular and Cellular Biology, National Centre for Biotechnology (CNB-CSIC), Campus de Cantoblanco, Darwin 3, 28049 Madrid, Spain; CIBERER, ISCIII, Madrid, Spain; CNRS, TAAM-CDTA UPS44, 3B rue de la Férollerie, CS 20057 45071 Orléans Cedex 2, France; Karolinska Center for Transgene Technologies, Comparative Medicine, Karolinska Institutet, von Eulers väg 4a, 171 77 Stockholm, Sweden; Biocenter Oulu, University of Oulu, Aapistie 5 A, 90220 Oulu, Finland; ICS France Institut Clinique de la Souris, PHENOMIN, ICS-MCI, CNRS, INSERM, Université de Strasbourg, 1 rue Laurent Fries, 67404 Illkirch, France; Institute of Experimental Genetics, Helmholtz Zentrum München—German Research Center for Environmental Health (GmbH), Neuherberg, Germany

**Keywords:** Cryopreservation, Mouse, PCR, Quality control (QC), 3R’s, Network of repositories

## Abstract

With the advent of modern developmental biology and molecular genetics, the scientific community has generated thousands of newly genetically altered strains of laboratory mice with the aim of elucidating gene function. To this end, a large group of Institutions which form the International Mouse Phenotyping Consortium is generating and phenotyping a knockout mouse strain for each of the ~20,000 protein-coding genes using the mutant ES cell resource produced by the International Knockout Mouse Consortium. These strains are made available to the research community via public repositories, mostly as cryopreserved sperm or embryos. To ensure the quality of this frozen resource there is a requirement that for each strain the frozen sperm/embryos are proven able to produce viable mutant progeny, before the live animal resource is removed from cages. Given the current requirement to generate live pups to demonstrate their mutant genotype, this quality control check necessitates the use and generation of many animals and requires considerable time, cage space, technical and economic resources. Here, we describe a simple and efficient method of genotyping pre-implantation stage blastocysts with significant ethical and economic advantages especially beneficial for current and future large-scale mouse mutagenesis projects.

## Introduction

The International Mouse Phenotyping Consortium (IMPC) (Ayadi et al. 2012; Brown and Moore 2012a, b; Laughlin et al. 2012; Ramírez-Solis et al. 2012; White et al. 2013) is a large scale international consortium whose aim is to generate and primary phenotype a knockout mouse strain for each of the ~20,000 protein-coding genes using the mutant ES cell resource produced by the International Knockout Mouse Consortium (IKMC) (Bradley et al. 2012; Skarnes et al. 2011). Cryopreservation strategies have been adopted for long-term storage of these and other research animal resources (Glenister et al. 1990). This facilitates their availability to the worldwide scientific community and provides resilience to potential catastrophic loss of a strain. To this end, several large centralised repositories have been established around the world, including the Infrafrontier/European Mutant Mouse Archive (EMMA) (INFRAFRONTIER Consortium 2014; Wilkinson et al. 2010), the KnockOut Mouse Project Repository (KOMP) (Lloyd 2011), the Jackson Laboratory Repository (Jax) (Ostermeier et al. 2008), The Center for Animal Resources and Development (CARD) (Nakagata and Yamamura 2009) and the Riken Bio Resource Center (Yoshiki et al. 2009), which provide cryopreserved material or live mice to receiving laboratories.

Ensuring a high level of quality control and validation of cryopreserved mouse germplasm is imperative. This is a long and expensive process that has to be performed for every batch of frozen material. Repositories of mutant strains therefore invest a significant amount of cost, time and resources to assess and secure the quality and vitality of cryopreserved sperm and embryo banks. This process also requires that many animals are bred and sacrificed to validate the freezing process and perform QC.

A typical validation will involve thawing cryopreserved sperm to ensure fertilisation post-thaw can be achieved at a predetermined level (>10 % of treated eggs at EMMA) and can produce viable embryos. This requires an in vitro fertilisation (IVF) procedure followed by surgical embryo transfer to pseudopregnant recipient females, pregnancy with births, and confirmation of the expected mutant genotypes from the resultant litter usually from tissue derived from an ear clip. Often, the genotyped mice have no additional use. The strain is considered secure when the quality control (validation process) is concluded.

Many animals need to be produced to support the development of the embryos to term, i.e. enough oestrus foster mice to be mated with vasectomised males to have suitable (vaginally-plugged) animals for the surgical embryo transfer. Typically, 2–3 transfers are performed per strain to be validated. Surgery must also be performed to generate the vasectomised males. Additionally, there can be a high repeat rate of the QC process if failed pregnancy and/or low birth rates result in a lack of sufficient pups from which to confirm the genotype.

We have successfully investigated a new simple and efficient methodology that involves the viability testing and genotyping directly on individual preimplantation blastocyst-stage embryos generated from fresh IVF or from frozen/thawed 2-cell stage embryos originally produced by IVF. This new approach has major ethical advantages by reducing greatly the number of animals used in the QC process. It also ensures that QC is processed faster, more robustly, and with significant reductions in cage space, technical resources, and overall cost.

## Materials and methods

### In vitro fertilisation (IVF) and embryo culture

For each batch of cryopreserved sperm, preimplantation embryos were generated by thawing a sample of the cryopreserved sperm and fertilising in vitro superovulated wildtype oocytes from the same genetic background (usually C57BL/6) as previously described (Behringer et al. 2014). To demonstrate the survival and fertilisation ability of the sperm post-thaw and to estimate the viability of the resource, the embryos were allowed to develop to the blastocyst stage in vitro or cryopreserved at the 2-cell stage and then thawed and cultured in KSOM media at a later date for 3–4 days to develop into blastocyst. For each strain, approximately 15 2-cell embryos were used to produce blastocysts. The probability of detecting a heterozygote is 99.99 % if analysing 15 embryos from a heterozygoye × wildtype cross.

### Blastocyst genotyping

The blastocysts are pooled into a 500 μl drop of M2 in a Petri dish. From here a single blastocyst is aspirated in a pulled Pasteur pipette in a minimal volume of M2 and placed directly into the bottom of a 0.2 ml PCR tube or in a well of a 96-well plate. This plate can be stored frozen at −20 °C.

#### Blastocyst genotyping conditions

##### DNA lysis

10 μl of lysis buffer PBND containing 0.1 mg/ml Proteinase K is added directly to each well containing a blastocyst and the plate placed in a Thermomixer with agitation at 56 °C for 30 min rising to 95 °C to for 10 min to inactivate the Proteinase K.

Samples are vortexed (10–15 s) and allowed to cool briefly then centrifuged for 1 min.

**PBND(**PCR Buffer Nonionic Detergents)

50 mM KCl

10 mM Tris–HCl (PH 8.3)

2.5 mM MgCl_2_

0.1 mg/ml gelatin

0.45 % v/v Nonidet P40 (NP40)

0.45 % v/v Tween 20

**Proteinase K** SIGMA-*ALDRICH*

G1N350 100 mg in 5.05 ml H_2_O

##### PCR amplification

Reaction conditions are shown below a PCR with specific primers and/or internal control primers, using 1.5 units of AmpliTaq Gold (other polymerases have also been successfully used including Invitrogen platinum Taq, Roche Taq polymerase, SuperTherm Taq polymerase and Phire Green Hot Start II DNA Polymerase).

Cycling conditions will vary depending on the annealing temperature of the primers and the gene to be amplified, and can vary between 35 and 60 cycles. **PCR MIX with specific primers:**

**Table:** 

dNTPS	(2 mM)	3 μl
PCR BUFFER 10×		3 μl
PRIMER 1	(0.5 μM/μl)	
PRIMER 2	(0.5 μM/μl)	
PRIMER 3	(0.5 μM/μl)	
H_2_O		
TAQ POLYMERASE	(5 U/μl)	0.3 μl
DNA	(5 μl of lysate)	5 μl
**TOTAL**		30 μl

**PCR MIX with specific and internal control primers:**

**Table:** 

dNTPS (2 mM)		3 μl
PCR BUFFER 10×		3 μl
PRIMER 1	(0.5 μM/μl)	
PRIMER 2	(0.5 μM/μl)	
PRIMER IL-6 For	(0.1 μM/μl)	
PRIMER IL-6 Rev	(0.1 μM/μl)	
H_2_O		
TAQ POLYMERASE	(5 U/μl)	0.3 μl
DNA	(5 μl of lysate)	5 μl
**TOTAL**		30 μl

*Selection of genes and primers that can be used as internal controls

**Interleukin 6 (IL-6) Amplified product170** **bp**

**IL-6 For** 5′-TTC CAT CCA GTT GCC TTC TTG G-3′

**IL-6 Rev** 5′-TTC TCA TTT CCA CGA TTT CCC AG-3′

**Kelch-like protein 18** (Klhl18) 524 bp

Klhl18_42066_For 5′ CCTGTGACAAGCAGTCTGAAGG

Klhl18_42066_Rev 5′ TGCTAGGGAGTGAATCTAGGGC

**Immunoglobulin heavy chain-joining region (**Igh-j) **290** **bp**

Igh-j For 5′ TGT-CCA-GGG-TCT-ATC-GGA-CT

Igh-j Rev 5′ GTT-TTT-CCT-CTG-TAC-CCG-AC

**Bradykinin receptor, beta 1 (B1 receptor gene) 340** **bp**

B1 For 5′ CTC-AGG-GAG-GCC-AGG-ATG-TG

B1 Rev 5′ TCA-GCG-GGG-TCA-TCA-AGG-CC

**Ventral anterior homeobox gene (Vax 1) 400** **bp**

VAX 1 For 5′ CGT-AAT-CAA-TTG-CAA-CAG-CGA-G

VAX 1 Rev 5′ AGA-AGG-AGG-GTG-GGA-AAA-GAA-G

**Gremlin 1 gene (Grem 1) 500** **bp**

Grem 1 For 5′ ATG-AAT-CGC-ACC-GCA-TAC-ACT-G

Grem 1 Rev 5′TCC-AAG-TCG-ATG-GAT-ATG-CAA-CG

**Retinoblastoma gene (Rb1) 650** **bp**

Rb1 For 5′ GGC-GTG-TGC-CAT-CAA-TG

Rb1 Rev 5′ AAC-TCA-AGG-GAG-ACC-TG

**Pyruvate kinase, muscle** Pkm 529 bp

Pkm For 5′ TTTGAGTAGCACCCACATAACCA

Pkm Rev 5′ CATGAAAAAGACCACCCCTGAAC

**ROSA 420 bp**

Rosa For 5′-ACT GGG ATC TTC GAA CTC TTT GGA C

Rosa Rev 5′-GATGTTGGGGCACTGCTCATTCACC

*(Bonaparte et al. 2013)

**DNTPS** (Deoxynucleoside Triphosphate Set PCR Grade) 4 × 25 µmol (250 µl) *Roche*

**PCR Buffer10× & 15** **mM MgCl**_**2**_*Applied Biosystems*

**AmpliTaq Gold DNA 250 Units, 5** **U/µl***Applied Biosystems*

### Strains do not fail QC due to pregnancy/birth failure

Analysis of collective repository QC data of frozen/thawed sperm obtained over a period of 4 years where IVF derived embryos were transferred to recipient females, showed that no frozen strains have failed the QC process due to the inability of preimplantation embryos produced by IVF to implant and develop to term, or because of a failure of the newborn pups to thrive. Table 1 shows that of a total of 918 strains cryopreserved, thawed and transferred to recipient females for QC, 30 failed to pass the frozen/thawed sperm QC. Of these 30, the largest proportion of failures (26) was due to fertilisation rates (i.e. development from zygote to 2-cell stage embryo) below an arbitrary 10 % rate of the number of oocytes used during the IVF. This does not necessarily mean that a strain could not be recovered, only that the operational threshold defined by the standard operation procedure (SOP) was not met. In fact, of these 26, 8 strains resulted in recovering live pups. The other four failed strains were due to a genotype mismatch caused by the use of the incorrect mice during the freezing. This data suggested that demonstration of IVF-derived embryos developing past the 2-cell to the blastocyst stage and which were correctly genotyped could be sufficient to provide a robust QC assessment without the need to transfer the embryos to recipient females.

**Table 1:** Showing the number of freeze attempts and the reason of failures in the QC process post thaw and IVF using IKMC derived EUCOMM/KOMP USD alleles on a C57Bl6/NTac genetic background

Strains	Number
Cryopreserved	918
QC-failed	30
Low fertilisation rate failures (<10 %)	26
Incorrect genotype failures	4

To date data from The Wellcome Trust Sanger Institute (WTSI) and The European Mouse Mutant Archive (EMMA) partners, show that failure to recover a line due to an inability to produce live offspring following thawing of cryopreserved sperm and IVF has never occurred

### Blastocysts can be genotyped in a robust manner

**Fig. 1:**
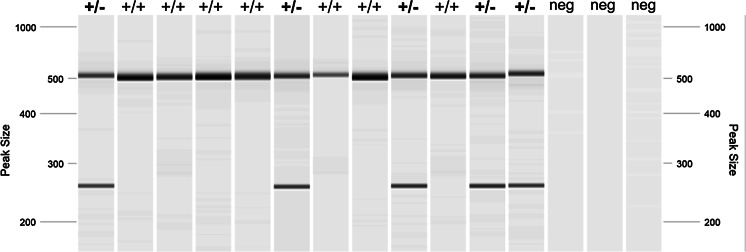
Blastocyst PCR reaction for Lrrc71^tm1a(KOMP)Wtsi^ using three primers in multiplex to amplify the WT (515 bp) and mutant (258 bp) alleles in the same reaction. Samples are visualised on a QIAxcel Advanced System (Qiagen). Genotype calls are shown above each sample lane and the last three lanes contain negative controls

We used PCR reactions to generate the genotypes from blastocyst stage embryos. Figure 1a, shows a strain example of gel electrophoresis of the PCR products demonstrating the genotypes obtained from blastocysts. Table 2 summarizes the experience across the multiple members of the Infrafrontier consortium. A total of 289 strains have undergone quality control by blastocyst PCR genotyping of which 283 (98 %) have passed QC. In terms of individual blastocysts, a total of 4450 have been analysed and 4053 (91 %) have successfully amplified DNA to demonstrate the genotype. A very few strains have failed to be successfully genotyped by blastocyst genotyping (2 %). These appear to be technical failures of the PCR possibly due to primer design or secondary structure making the region difficult to amplify, or where one assay works but another fails making the final genotype call ambiguous.

**Table 2:** Overall and cross centre results using blastocyst genotyping

Centre	Strains			Blastocysts		
	QC’ed	Pass	Success rate	QC’ed	Pass	Success rate
CNB^3,4^	17	17	100	325	325	100
CNR^1^	62	61	98	1104	1070	97
CNRS^5^	21	20	95	240	203	84
HMGU^9^	14	14	100	406	386	95
ICS^8^	61	60	98	718	639	89
KI^6^	12	12	100	97	96	99
Oulu^7^	18	16	89	235	209	89
WTSI^2^	84	83	99	1325	1125	85
Totals	289	283	98	4450	4053	91

## Discussion

We tested a much improved quality control method to determine the ability to recover a mutant mouse strain which has been stored in a cryopreserved form. Two conditions need to be met to apply this methodology a) a large proportion of fertilised embryos developed from IVF in vitro should survive to produce blastocysts and b) a reliable method to genotype blastocysts must be available.

Instead of transferring IVF generated 2-cell stage embryos into recipient females to allow development to term and genotyping the resultant pups, our approach relies in vitro culture of the IVF-derived embryos to the blastocyst stage and genotyping of these embryos. Only 15 embryos per strain are required to perform the blastocyst genotyping QC with a 99.99 % probability, as compared to the 40–60 embryos (i.e. 2–3 embryo transfers) necessary when using the current QC protocol for pup generation. An additional advantage is that more fertilization events (i.e. embryos) can be genotyped because losses due to lost pregnancies (~20 %) or other development issues which normally reduce the number of surviving pups to 30 % are avoided.

The blastocyst genotyping approach is flexible and can be used for all kinds of mutations including those generated by recent genome editing techniques such as Crispr/Cas9 that can be discriminated by one or a limited number of PCR reactions (the DNA extracted from a blastocyst is sufficient for 2–3 PCR reactions). The method can be applied to any large or small scale repositories that maintain sperm or embryos as frozen stocks. Given the size of the blastocyst, caution needs to be exercised in the embryo manipulations prior to lysis to avoid sample loss.

This shortens the QC process by approximately 2 months and saves a significant amount of resources whilst providing a clear ethical improvement compatible with 3R’s (Reduction, Refinement, Replacement) principle championed by Russel and Burch (1959) since it reduces the number of animals bred, subjected to surgery and sacrificed for genotyping. We have calculated that we would save on average 14 animals per strain across all contributing centres if using blastocyst genotyping to confirm the QC of the cryopreservation process when compared to conventional methods. Animals would be saved at various points in the QC process including recipient foster mothers (2), vasectomised males (1), pups born to be genotyped (8), and females used as oocyte donors (3). For a large-scale project like IMPC, an estimated 5000 strains will have been deposited by the end of 2016 (Phase 1) (Brown and Moore 2012b) Using blastocysts to verify the QC process for cryopreserving this number of lines would save an estimated 70,000 animals over current methods of genotyping pups.

We also calculated how many cage-weeks using this methodology would save. On average we save 14 cage-weeks per line using traditional genotyping methods (Recipient cages pre and post embryo transfer, breeding cages for stocks, female donor cages for superovulation, and cages to house vasectomised males). Therefore across the life of the project calculating 5000 strains generated for the IMPC in phase 1 of the project this would save 70,000 cage weeks across a 5 year period.

Other potential uses of blastocyst genotyping surround new genome editing technologies such as Crispr/Cas9 (Wang et al. 2013). Culturing of embryos to blastocyst of a few experimentally injected embryos will allow a first look to determine the efficiency of the gRNA’s and Crispr materials in generating your desired mutation. Blastocyst genotyping may also be used as a first experimental step with Crispr/Cas9 in evaluation and optimisation of experimental design in generating mutants with more complex alleles for which efficiencies currently remain low with this technology, such as large insertions in homologous recombination.

In summary, using blastocyst genotyping instead of conventional methods leads to significant reductions in animal welfare concerns, processing time, technical requirements, and cost. Therefore, we would like to propose the blastocyst genotyping method as the preferred protocol for QC purposes to validate the correct cryopreservation of mouse strains in world-wide large mouse embryo and sperm banks.
